# Sustainability of Animal Production Chains: Alternative Protein Sources as an Ecological Driver in Animal Feeding: A Review

**DOI:** 10.3390/ani15223245

**Published:** 2025-11-08

**Authors:** Massimiliano Lanza, Marco Battelli, Luigi Gallo, Francesca Soglia, Fulvia Bovera, Francesco Giunta, Riccardo Primi, Luisa Biondi, Diana Giannuzzi, Marco Zampiga, Nicola Francesco Addeo, Antonello Cannas, Pier Paolo Danieli, Bruno Ronchi, Gianni Matteo Crovetto

**Affiliations:** 1 Department of Agriculture, Food and Environment (Di3A), University of Catania, 95123 Catania, Italyluisa.biondi@unict.it (L.B.); 2 Department of Agricultural and Environmental Sciences (DiSAA), University of Milan, 20133 Milan, Italy; matteo.crovetto@unimi.it; 3 Department of Agronomy, Food, Natural Resources, Animals and Environment (DAFNAE), University of Padova, 35020 Padova, Italy; luigi.gallo@unipd.it (L.G.); diana.giannuzzi@unipd.it (D.G.); 4 Department of Agricultural and Food Sciences (DISTAL), Alma Mater Studiorum—University of Bologna, 40127 Bologna, Italy; francesca.soglia2@unibo.it (F.S.); marco.zampiga2@unibo.it (M.Z.); 5 Department of Veterinary Medicine and Animal Production (DMVPA), University of Napoli Federico II, 80137 Napoli, Italy; bovera@unina.it (F.B.); nicolafrancesco.addeo@unina.it (N.F.A.); 6 Department of Agricultural Sciences, University of Sassari, 07100 Sassari, Italy; giunta@uniss.it (F.G.); cannas@uniss.it (A.C.); 7 Department of Agriculture and Forest Sciences (DAFNE), University of Tuscia, 01100 Viterbo, Italy; primi@unitus.it (R.P.); danieli@unitus.it (P.P.D.); ronchi@unitus.it (B.R.)

**Keywords:** novel feeds, alternative legumes, microalgae, insects, camelina sativa byproducts, soybean silage, *tef*

## Abstract

Sustainability of animal production requires reducing reliance on soybean meal by identifying viable alternative protein sources. Within the framework of the Italian Agritech National Research Center, seven Italian research groups collaborated to evaluate unconventional feed ingredients and their effects on animal performance and product quality. Alternative legume seeds (peas, chickpeas, faba bean, and lupins) can partially or completely replace soybean meal without impairing productivity, while enhancing product health value and shelf-life through bioactive compounds. Microalgae (*Chlorella*, *Spirulina*) improved carotenoid content, antioxidant activity, fatty acid profile, and cholesterol levels in poultry products, with limited effects in pigs. Insects supported optimal growth in fish at 25–30% inclusion, whereas maximum recommended levels are 15% in broilers and 24% in laying hens to sustain growth, egg production, and quality. Camelina by-products are suitable for poultry diets at up to 5–10%, beyond which performance declines. Whole-plant soybean silage, tef (*Eragrostis tef*), and triticale–lupin intercropping represent promising protein-rich resources for ruminants, provided diets maintain balanced protein-to-energy ratios, adequate fibre characteristics, and appropriate harvest timing under drought-prone conditions. Collectively, these findings highlight the potential of diverse protein sources to improve the sustainability of livestock systems while preserving productivity and enhancing the nutritional quality of animal-derived foods.

## Simple Summary

Making animal production more sustainable requires reducing the heavy reliance on soybean meal, which is often imported and has a significant environmental footprint. This review explores a range of alternative protein sources that could partly replace soybean meal in livestock diets without harming animal growth, productivity, or product quality. Legume seeds such as peas, chickpeas, faba beans, and lupins can be used successfully and may even enhance the nutritional value and shelf-life of meat, milk, and eggs. Microalgae like *Chlorella* and *Spirulina* can improve the levels of healthy fatty acids and antioxidants in poultry products, while insects show promise as feed for fish, poultry, and laying hens if used at the right inclusion levels. Camelina by-products can be included in poultry diets, though only at moderate amounts to avoid performance losses. For ruminants, whole-plant soybean silage, tef (*Eragrostis tef*), and lupin–triticale intercropping are valuable options, especially under dry conditions, provided that diets remain balanced in protein and fibre. Together, these alternative protein sources represent practical strategies to reduce dependence on soybean meal, improve the nutritional profile of animal-derived foods, and move livestock production toward greater environmental sustainability.

## 1. Introduction

Global food demand (including both plant- and animal-based products) is projected to increase by 35–56% by 2050 compared with 2010 levels, and by up to 62% when climate change effects are considered [1]. This trend is mainly driven by population growth and the rising consumption of animal-derived foods, as highlighted by the Food and Agriculture Organization (FAO) [2].

One of the big challenges for livestock production chains is to enhance sustainability by mitigating environmental impacts such as land use change for feed production, greenhouse gas (GHG) emissions, water overuse, eutrophication and acidification processes, and loss of biodiversity. These issues gained wide public attention after the publication of the FAO report “Livestock’s Long Shadow: Environmental Issues and Options” in 2006 [3]. More recently, concerns have expanded to include feed–food competition [4,5], and antimicrobial resistance [6], while precision livestock farming technologies have emerged as valuable tools to address both environmental and social aspects of sustainability [7].

Within this global framework, the livestock sector in the European Union (EU) faces an additional challenge: the limited availability of high-protein feed sources. The EU is only about 28% self-sufficient in high-protein (30–50%) feed ingredients, mainly represented by oilseed meals [8]. Consequently, European livestock production relies heavily on imported soybean meal, exposing the sector to price volatility and supply risks in global markets. Moreover, soybean production itself raises environmental sustainability concerns, particularly in exporting countries where expansion often occurs through deforestation or conversion of natural ecosystems [9,10]. The situation is further complicated by the prevalence of genetically modified (GM) soybean—accounting for about 105 million hectares and 80% of global soybean production in 2017 [11]—which is banned in organic livestock systems [12]. These factors underline the urgency of identifying and developing alternative, locally available protein sources to enhance both the environmental and economic resilience of European livestock systems. This is of particular importance in the EU countries, where the trend in organic livestock production is growing. For example, bovines organically reared in EU were 5.5% of the total herd in 2018, and became 7.2% of the total herd in 2022 [13].

Within this wide context, the Italian Agritech National Research Center (https://agritechcenter.it/, accessed on 5 November 2025) involved seven Italian research groups in the “Alternative protein sources for animal nutrition” topic. They focused their attention on some specific protein source or animal species according to their scientific backgrounds: they chose to explore the state of the art on the use of legume seeds, microalgae, insects, and camelina by-products as an alternative to soybean meal in concentrate for monogastrics or fish species. Specifically for ruminant husbandry, where the farm-based forage production usually represents the economic pillar of the enterprise, they chose to explore the chance to increase the protein self-sufficiency, focusing on the high-quality forage production to replace, at least partially, soybean meal in livestock diets. The attention has been focused on soybean as silage fodder and on tef. This review summarizes some potential alternative protein sources in animal feeding as a favourable option to guarantee sustainability to livestock production systems, focusing on their effects on animal performance and product quality or on enteric fermentation.

## 2. Materials and Methods

A thorough screening of the literature was carried out to evaluate the effects of total or partial replacement of soybean meal with alternative protein sources, with the aim to test if it can represent a valid strategy to improve the sustainability of animal production chains maintaining the profitability in terms of animal performance and product quality.

The literature search was performed by using repositories such as Web of Science, Scopus, ScienceDirect, and Google Scholar databases on selected search strings, such as the following: “alternative protein sources AND animal nutrition”, “alternative legume seeds AND ruminants”, “alternative legume seeds AND monogastrics, “microalgae AND animal nutrition”, “insect AND animal feeding”, “underexploited plants AND animal nutrition”, “TEF as alternative forages AND cattle feeding”, “Soybean as forage AND animal nutrition”, “Soybean silage AND ruminant nutrition”, and “Camelina cake AND animal feeding”. The review process has taken into consideration research findings in a timespan mostly covered from 2000 to the present, and applied to ruminants, monogastrics, and fish.

## 3. Alternative Protein Sources as Concentrates

### 3.1. Legume Seeds

Nowadays, in Europe, legume production is still marginal, covering less than 3% of arable land [14], although legumes show beneficial effects in animal and human nutrition, in crop rotations, and for the ecosystem in general [15,16].

Legume grains (namely pulses) are being considered in competition between human and animal feeding; nonetheless, they could represent a valid option to support the protein requirements of livestock in place of soybeans. Moreover, the ban of GMO soybeans as a protein source in organic farming systems boosted the utilization of grain legumes together as in low-input systems [17].

In the EU regulatory schemes, by simplification, legumes were divided into two large categories: protein crops (e.g., peas, faba bean, lupins, soy, alfalfa, etc.) mainly intended for livestock, and legumes from grain (e.g., beans, lentils, peas, chickpeas, etc.) mainly intended for human use.

#### 3.1.1. Nutritional Characteristics and Factors of Influence

Table 1 reports an average nutritional characterization of the most used legume seeds in animal nutrition. Faba bean (*Vicia faba*) and the protein pea (*Pisum sativum*) are the main legume grains used as feed, generally processed (crushing, heat treatments) to inactivate potential antinutritional factors and then mixed for the composition of the feed. Faba bean, pea, and chickpeas show a protein content around 23–29% on DM, while in lupins the protein level is higher [15], ranging between 35 and 40% in *Lupinus albus*, *L. luteus*, and *L. angustifolius* [18].

Grain legumes contain seed protein that is relatively deficient in the sulphur amino acids, cysteine and methionine. Lupin usually presents a high level of arginine [30], which has a positive role in the reduction of embryo losses in the early uterine stages of development, both in ruminants and in monogastric animals [31]. In general, untreated legume seeds show a high degradability in the rumen that can be reduced through heat treatments [32]. A limited rumen escape fraction of the crude protein (CP) of lupin has been detected [18]. A difference among faba bean, peas, chickpeas vs. lupins concerns the starch content, much higher in the former compared to the latter (30–40% vs. 1–5% on DM, respectively). Lupins, however, usually present high content of non-fibre carbohydrates (28–30% DM), made by complex non-starch polysaccharides, and an average lipid content of 6.5–10% [18]. The gross energy contents are quite similar among legume grains (18–21 MJ/kg DM). Metabolizable energy (ME) contents of legume grains are in the range 11.8–16.5 MJ/kg DM.

Several bioactive compounds were detected in pulses as reported by Muzquiz et al. [33] (Table 2).

Their concentration can be influenced to a certain extent by genotype, climatic conditions, and agronomic techniques, thus increasing the variability in quality [44]. These compounds can be mainly classified under phytochemicals such as phenolic compounds (flavonoids, anthocyanins), saponins, carotenoids, and tocopherols [45]. Moreover, phytosterols, oligosaccharides, and resistant starch were also detected as bioactive constituents in pulses [46]. Among phenolic compounds of legume seeds, phenolic acids, flavonoids, and condensed tannins play a major role. In particular, tannins have a controversial dose-dependent role reducing voluntary feed intake and nutrient digestibility at high concentration [47] while improving ruminant nutrition and healthiness at medium–low doses [48]. Legume grains contained variable proportions of tannins, and high- and low-tannin varieties are available with different nutritional values according to animal species.

#### 3.1.2. Feeding Studies

Low-tannin content generally results in higher protein and energy digestibility for monogastric animals, either in swine or in poultry [49]. Ruminants are more tolerant of tannins compared to monogastric animals. No detrimental effects on dairy milk production and composition, when faba bean was added at a proportion of 17% of dietary DM and when tannin content was 0.42% DM, have been observed [50]. In grazing cows, the supplementation with faba bean tended to increase milk yield when using a variety with a high level of polyphenols (total polyphenols 16.4 mg of gallic acid equivalent/g DM), without relevant effects on milk quality or grazing behaviour [51]. Also, in lambs, the total replacement of soybean meal with faba bean (38% on a DM basis) did not impair growth performance and meat quality [52]. The inclusion of low- or high-tannin faba bean varieties in diets supplied to pigs and poultry showed controversial results, and it was emphasized that the main role of amino acid balance that could reduce the potential negative effects of high tannin intake [49]. The inclusion of peas (24 or 40% on a fed basis) in concentrates supplied to growing lambs improved meat fatty acid profile through an increased level of C18:3 linolenic and total n-3 polyunsaturated fatty acids (PUFAs) compared to lambs fed on concentrates including faba bean or soybean meal as main protein sources [52,53]. The replacement of soybean with 20% peas did not negatively affect performance and meat quality in heavy pigs [54]. Overall, the use of peas in swine nutrition should consider the sulphur amino acid deficiency and the presence of secondary plant metabolites, which can be both easily overcome through association with cereals or by adding synthetic amino acids to the diets, together with improving the use of legume cultivars with low content of secondary compounds [22]. Also, lupins were hugely investigated in different animal species and overall did not significantly change animal performances and product quality either as the sole replacers of soybean or combined with other alternative protein sources [55,56,57,58,59]. Two reviews on the use of lupin in livestock feeding are worth mentioning: Petterson [30] and White et al. [18]. Overall, the studies carried out on ruminants showed a positive effect of lupin when used as a substitute for cereals, for its higher energy concentration, due to its crude fat content, and lower content of non-fibrous carbohydrates (NFCs), which in cereals are often causative of sub-acute acidosis and milk fat depression. Regarding lupin utilization as a protein source, the comparisons made with soybean meal were associated with no differences in milk production but reduced milk protein content, possibly for the low methionine and lysine concentration of lupin. Most of the ruminant studies [16,25] were carried out on animals of intermediate productivity and with relatively high concentrations of dietary CP, conditions that do not properly challenge the ability of lupin to substitute high-quality proteins, as soybean meal. It would be advisable to assess the maximum inclusion level of lupin seeds in highly productive ruminants. As for meat ruminant production, Lestingi et al. [60] reported a negative effect on fatty acid profile in lambs’ meat when lupins were used alone, while not when combined with peas. In swine, the use of lupins was limited by alkaloids and non-starch polysaccharides that impaired digestibility and gut efficiency. Nevertheless, the selection of varieties with low antinutritional factors combined with methionine, tryptophan, and threonine supplementation can counteract these issues [61]. Strakovà et al. [62] observed that the substitution of 50% of soy protein for white lupin protein in feed mixtures did not negatively affect the health status of the laying hens. In broiler ducks, *L. luteus* supplementation in total replacement of soybean meal improved meat colour and fatty acid profile but impaired collagen content [63], while in broiler chicken, when dehulled and micronized, *L. albus* improved fatty acid profile [28]. Cowpea (*Vigna unguiculata*) and chickpea can be used as a replacement for soybean meal in broiler chicken diets, at inclusion levels up to 200 g/kg [64].

#### 3.1.3. Focus on Lupin Growing in an Intercropping System

In a Mediterranean environment, lupin grows as a rain-fed crop during the autumn–winter period and is therefore more economically convenient than soybean, which can only be grown as an irrigated crop during the spring and summer. Notwithstanding the ecosystem services that grain legumes produce in agro-ecosystems [65], lupin is not widely cultivated in the EU, where in 2019 it occupied approximately 120,000 ha [66], with a relative grain production that is about 18% of the world’s production [66]. One of the causes of the poor cultivation of lupin in the EU is the high variability of the grain yield; FAOSTAT [66] shows 1.8 and 2.4 tons of grain per hectare in Italy and France, respectively. This variability is mainly associated with a high susceptibility to biotic and abiotic stresses [67] and a poor adaptability to calcareous soils [68].

Intercropping is the simultaneous growth of two or more crops in the same field [69]. It offers various advantages measurable in yield and yield stability, particularly under low-input conditions [69,70] and, in general, in all the agroecosystems in which nutrient availability, pests, and diseases [71] limit the yield of each companion crop. Another way in which intercropping leads to improved yields compared to sole grain legume crops is weed control [70].

Among the possible companions for legume crops, triticale (× *Triticosecale* Wittmack) is an outstanding species thanks to its total biomass and grain yield production and its adaptation to low soil pH, water-logging, and calcareous soils [72]. The greater competitiveness of the cereal in acquiring the soil nitrogen in comparison with the companion grain legume crops increases the rates of nitrogen fixation, and hence the role of N_2_ fixation in intercropping over legumes grown in rotation with cereals [73,74]. Another advantage deriving from intercropping grain legumes with cereals comes from the reduction of nitrogen losses through both leaching [75] and N_2_O emission [76] in comparison with the sole grain legume crop. Lupinus is a phosphorus-mobilizing species [77], which can increase the phosphorus uptake by the companion cereal crop [78], while cereals liberate Fe and Zn, increasing their uptake by the grain legumes companion crop [79,80,81]. Moreover, the slow canopy development of lupin makes the crop uncompetitive against weeds [82], but the high soil nitrogen acquisition by an intercropped triticale can reduce the weed biomass at lupin flowering by 63% [70], and consequently, the use of agrochemicals for weed control. Furthermore, some studies combined triticale and lupin to produce forages, mainly whole crop silages [83,84]; the white lupin (*Lupinus albus*), in particular, can be successfully ensiled as a whole crop [85]. The quality of silages obtained varied depending on the proportion between triticale and lupin and weeds at harvest and DM of the herbage, but in most studies, high ammonia values and difficulties in decreasing the pH of the silage were observed for these mixtures. It is well-known that a low DM content of the herbage at harvesting can be resolved by adopting pre-wilting before ensiling, a simple technical tool for overcoming these issues and obtaining good quality silage. Triticale and lupin mixed silages were used to substitute grass silages, with no detrimental effects on animal average daily gain and meat quality of beef cattle [83,84].

In conclusion, the intercropping of triticale and lupin can therefore be an interesting solution for increasing protein self-production within a livestock farm, whether it produces grains for concentrate, to reduce the use of soybean meal, or whether it produces high-quality forages, to reduce the concentrate-to-forage ratio. Moreover, the production of a mixed concentrate (triticale and lupin grains) or a mixed herbage supports the production of a balanced feed in terms of carbohydrates and protein proportion [86].

#### 3.1.4. The Challenge of Legume Seeds as Feed Ingredients

In perspective, replacing soybeans with legume seeds in concentrates fed to livestock should be reevaluated in terms of sustainability and potential added value of animal products linked to the transfer of some bioactive compounds naturally present in legumes [46]. Legume crops may exert a fundamental role in the crop rotation practices, especially in organic and agro-ecological farms, representing a valuable tool for improving livestock sustainability systems while reducing the dependence on imported protein sources. Literature analysis generally did not show detrimental effects either in terms of animal performance or product quality when legume seeds were partially or totally replaced by soybean. In terms of secondary compounds, the dose-effect plays a role in terms of impairing feed intake and digestibility, while more studies are needed to test their potential effects on the oxidative stability of animal products linked to the antioxidant power of most of them.

### 3.2. Microalgae: Chlorella sp. And Spirulina sp.

Microscopic aquatic organisms, referred to as microalgae, constitute a composite group of O_2_-evolving photosynthetic microorganisms, comprising prokaryotic cyanobacteria and eukaryotic members [87]. These microorganisms can produce protein-rich feed, while showing better CO_2_ fixation efficiency, absorption of solar energy radiation, and nutrient uptake than terrestrial plants [88].

Their microscopic nature offers several advantages compared to their macroscopic counterparts, including simplified genetic manipulation, streamlined scaling processes, and generally higher protein content [89]. The global production of microalgae currently stands at approximately 50,000 tons per year [90]. *Chlorella* sp. and *Spirulina* (Arthrospira) sp. together contribute to more than 90% of the world’s microalgal biomass production [91], primarily devoted to human consumption.

Currently, the *Chlorella* genus encompasses 14 species, with *C. vulgaris* and *C. sorokiniana* emerging for use as feedstock due to their favourable characteristics, including fatty acid composition, rapid growth, high productivity, and valuable biomass content [92]. *Spirulina* sp. and *Arthrospira* sp. consist of cyanobacterial species that share significant similarities, although they belong to taxonomically separate genera. Over time, many species previously classified as *Spirulina* sp. have been reclassified under the *Arthrospira* sp. genus, but they continue to be marketed with the commercial name of Spirulina [93]. Among them, *Arthrospira maxima*, *Arthrospira fusiformis*, and *Arthrospira platensis* are commonly employed in large-scale cultivation [94]. Henceforth, we will use the term Spirulina to collectively represent both *Spirulina* sp. and *Arthrospira* sp.

#### 3.2.1. Nutritional Characteristics

Microalgae have gained recognition for being nutrient-rich compounds. Both the *Chlorella* and *Spirulina* genera are known for their high protein content, which ranges between 50 and 70% of DM [95]; moreover, they present an excellent balance of essential amino acids [96], superior to that of soybean [97]. Microalgae also produce bioactive peptides with antioxidative, antihypertensive, anticoagulative, antitumor, and immune-stimulating properties [98].

Carbohydrates account for 15 to 25% of *Spirulina* DM, and glucose, rhamnose, xylose, and mannose are the most abundant fractions [95]. Microalgal biomass lacks hemicellulose and lignin and, in some species, also cellulose, as they have no cell walls [99]. This attribute makes *Spirulina* sp. more attractive than *Chlorella* sp., which has cellulosic cell walls and consequently lower digestibility [95]. Moreover, microalgae contain several polysaccharides with antioxidant properties [100].

Lipid content in *Spirulina* ranges between 4 and 10% of DM, with a substantial quantity (between 25 and 60% of total fatty acids) of PUFA [95].

Phosphorus, potassium, and calcium are highly prevalent in microalgae [95]. Moreover, they contain a huge amount of iron compared to other vegetables, and the absence of phytates and oxalates may support its absorption. Microalgae are rich in carotenoids, mostly as beta-carotene and cryptoxanthin, easily converted in mammals into vitamin A without causing cumulative toxicity [101]. Microalgae also contain antioxidant tocopherols (15 to 190 mg/g) and four times more vitamin B12 than the raw liver.

In addition, microalgae are an unexplored source of compounds such as lipoproteins, sterols, and alkaloids, substances that may exert anti-inflammatory, anti-atherogenic, anti-cancer, and antioxidative effects [102].

#### 3.2.2. Microalgae in Monogastric Nutrition

To date, approximately 30% of the global microalgae production is directed to the feed industries [103]. *Chlorella* and *Spirulina* are widely employed as feed supplements in various livestock species, harnessing their rich nutritional compounds to enhance immune response, antiviral and antibacterial action, disease resistance, and promote fertility and gut function. Instead, the effectiveness in using microalgae as feed ingredients relies on their nutritive value and the animal’s adaptation to those ingredients. Due to their nutritional characteristics, they may represent a viable alternative to protein feedstuffs such as soybean [104] for the livestock species considered in this section. However, differences in livestock species and the dietary content of microalgae resulted in contrasting effects on animal performance. A summary of recent findings by species follows hereafter (Table 3).

##### Poultry

The use of dried and/or defatted *Arthrospira* spp. and *Chlorella* spp. microalgae as a protein source in poultry nutrition has been evaluated in several studies. *Arthrospira* spp. is used in poultry feed as a partial replacement, typically up to a maximum of 20%, of conventional proteins, also to enhance the colour of skin, meat, and egg yolks [104]. Most of the studies conducted so far have tested *Chlorella* at very low dietary inclusion levels (i.e., below 2–3%), thus considering it more as a feed additive rather than a main protein ingredient [123]. Such an approach could be justified by the market price of this microalga and by its excellent nutritional profile, especially in terms of bioactive compounds content, which can positively affect animal performance and health even at low dosages [124,125].

Growing chicken and broiler: The inclusion of different levels of *Spirulina* in the diet exerted no effects on growth performance of broiler chickens [113,114,119] and carcass performance compared to conventional diets, but improved the apparent ileal amino acid digestibility [114]. More recently, Zampiga et al. [126] investigated the effects of the substitution of dietary soybean with graded dosages of *Arthrospira* spp. meal (5, 10, and 15%) during the first stages of the rearing cycle of broiler chickens. The results indicated that microalgae inclusion linearly reduced body weight and increased feed conversion ratio at 22 days. However, the administration of a soybean-based diet from 23 days to slaughter (47 days) allowed the birds receiving 5% *Spirulina* up to 22 days to recover overall productivity. Mullenix et al. [127], testing the effects of the dietary inclusion of 10% *Spirulina* in low crude protein diets from 15 days onwards, observed a slight reduction in body weight gain and a decreased feed efficiency in female chickens if compared to those fed the same low crude protein diet without *Spirulina*; such an effect was less pronounced in male chickens. One of the most consistent outcomes of *Arthrospira* on chicken meat quality is the capacity to modify skin and meat colour parameters [113,127,128]. Meat from *Spirulina*-fed broilers was more tender and softer, had greater pH and water-holding capacity, was redder, darker, and more yellow, and had increased umami and chicken flavour [115,116]. In general, some positive effects on growth performance of broiler chickens can be observed when *C. vulgaris* is included at dosages up to 1% [117]. Only a few studies have investigated the effects of *C. vulgaris* administration at higher dosages in broiler chicken diets, in partial replacement of soybean meal. The inclusion of 10% *C. vulgaris* (either alone or in association with exogenous enzymes that were supposed to disrupt cell wall integrity and thus to increase nutrient availability) from 21 to 35 days of bird age did not affect the productive performance of broilers and slightly improved meat quality traits and lipid nutritional profile [129]. Boskovic Cabrol et al. [123] incorporated *C. vulgaris* into starter and grower diets at 10, 15, and 20%. The broilers receiving the diet with the lowest dosage (i.e., 10%) performed similarly to those fed the soybean-based diet; however, when the microalga meal was provided at higher levels, final body weight, weight gain, and feed consumption were significantly compromised.

Laying hens: *Spirulina* inclusion in laying hen diets at 1.5, 2.0, and 2.5% did not substantially modify the egg production traits of Hy-line hens at 63 weeks of age, although it increased egg yolk pigmentation [119]. Similarly, Omri et al. [130] observed an increase in egg yolk redness, a reduction in yellowness, and no effects on performance traits when *Spirulina* was tested at dosages up to 2.5%. Tufarelli et al. [131] reported no adverse effects of 2% *Spirulina* administration on the productive performance of laying hens and observed an increased eggshell thickness, breaking strength, and yolk colour while reducing the cholesterol content. Recently, Al-Otaibi et al. [132] evaluated the effects of feeding laying hens diets containing 0, 3, 6, or 9% *Spirulina platensis* under either thermoneutral or cyclic heat stress conditions. The Authors concluded that *Spirulina* could relieve the negative effects of thermal stress on egg production traits, particularly when administered at the highest tested dosage. The supplementation of fermented *C. vulgaris* (0.1–0.2%) enhanced egg production, egg yolk colour, and Haugh index [133]. Recently, Panaite et al. [134], evaluating the partial substitution of soybean meal in laying hen diets with 2% *Chlorella*, observed an increase in feed conversion ratio and egg weight in response to its administration. As far as product quality is concerned, the dietary treatment improved β-carotene and cholesterol contents, antioxidant capacity, yolk colour, and determined a remarkable increase in the amount of n-3 polyunsaturated fatty acids in the yolk, resulting in a substantial reduction in the n-6/n-3 ratio. Improvements in yolk colour were also reported by Kim et al. [135], who tested *C. vulgaris* at very low dietary dosages (i.e., 0.5%). Similarly, the use of a selenium-enriched *Chlorella* at 0.12% was reported to increase egg deposition rate, egg weight, selenium content in both albumen and yolk, albumen weight, and Haugh index [136].

##### Pigs

In pigs, most studies have included *Spirulina* sp. and *Chlorella* sp. as dietary supplements in minimal amounts, revealing no or minimal changes in production traits and meat quality. In weaning pigs, microalgae showed no changes in growth performance, but especially *Chlorella* improved intestinal development without compromising nutrient digestibility [105,106,137]. The supplementation of *Spirulina* to suckling piglets increased weaning weight and reduced the incidence of diarrhea during the first two weeks following weaning, without enhancing digestive health [107]. Conversely, Martins et al. [108] indicated a drop in growth performance in piglets when 10% of *Spirulina* was included in the diet, mainly because of a reduced protein digestibility due to an increase in digesta viscosity. Altmann et al. [111] replaced 50 to 75% of soybean with *Spirulina* in diets for barrows without effects on carcass characteristics, but they observed an increase in PUFA meat content. Similarly, Coelho et al. [109,110] reported that the dietary inclusion of *Chlorella* (5%) did not affect growth rate, carcass, and meat quality traits of finishing pigs, but increased the hepatic content of n-3 PUFA. Last, supplementation with *Spirulina* in the diet of pregnant and lactating sows did not impact the carcass and meat quality of the offspring [112].

##### Rabbits

In recent years, few studies have dealt with the effects of dietary inclusion of microalgae in rabbit diets. The inclusion of *Chlorella* (200 to 500 mg/kg BW) in prepuberal rabbits increased weight gains and feed utilization [120]. Also, El Basuini et al. [122] found positive effects of a dietary inclusion of *Spirulina* or *Chlorella* on the performance of growing rabbits, whereas Abdelnour et al. [121] did not.

#### 3.2.3. The Challenge of Microalgae as Feed Ingredients

The utilization of microalgae as feedstock holds strategic importance, presenting potential for competitive advantages. Beyond providing a broad spectrum of excellent compounds, microalgae, unlike soybeans, do not necessitate arable land for cultivation, being produced in water culture or raceway ponds [103]. Hence, their use could contribute to agricultural sustainability, addressing the need to reduce land degradation and water deprivation, and mitigating the competition with the human food crops [138]. Current drawbacks are represented by the potential of microalgae to accumulate heavy metals, due to intracellular detoxification mechanisms [139]. Grosshagauer et al. [139] highlighted variable heavy metal accumulation in *Spirulina* biomass, with occasional exceedance of the EU limit for lead (3 mg/kg) and detectable inorganic arsenic in some commercial products, whereas mercury and cadmium generally remain within regulatory thresholds. These findings underscore the importance of strict cultivation management and standardized analytical monitoring to ensure product safety.

Furthermore, the current cost per kilogram, partly concerning the drying and conditioning of algae biomass, poses a challenge within the livestock production economy, thereby limiting their widespread commercialization as feed. Therefore, strategies able to lower production costs while maintaining quality standards of microalgae should be a research priority for the next year [138]. Last concern is related to the potentially low digestibility and bioavailability of microalgae nutrients. Feed processing techniques can address this issue, highlighting promising results, but caution must be paid to prevent nutrient dispersion or nutrient imbalance [140].

### 3.3. Insects

Insects have been extensively studied as a concentrated source of protein for animal feed, often in the form of meals. For monogastric animals such as poultry, fish, swine, and even pets like dogs and cats, insect meals can serve as a viable alternative to traditional protein sources like soybean meal and fish meal. Recently, insects have emerged as the most promising and sustainable source of animal protein, primarily due to their high nutritional value. Many insect species showed favourable conversion factors and productivity, rapid life cycles, resulting in high-quality proteins, fatty acids, vitamins, and functional compounds that can be easily assimilated. Moreover, insects can efficiently convert organic waste into high-quality proteins for animal feeding, thereby reducing the costs and burdens for waste management while improving resource utilization and nutrient recovery [141].

In Europe, the European Commission has authorized the use of farm insect meals as feedstuff in aquaculture (European Commission, regulation 893/2017) and recently in commercial poultry and pig nutrition (European Commission, regulation 1372/2021), insects processed animal proteins (PAPs) that can be obtained from the following seven species: Black Soldier Fly (*Hermetia illucens*), Common Housefly (*Musca domestica*), Yellow Mealworm (*Tenebrio molitor*), Lesser Mealworm (*Alphitobius diaperinus*), House cricket (*Acheta domesticus*), Banded cricket (*Gryllodes sigillatus*) and Field Cricket (*Gryllus assimilis*).

#### 3.3.1. Insects in Aquaculture: Performance and Product Quality

Rising prices of fish meal and fish oil have prompted the search for sustainable alternatives for aquaculture feed. Insects, which are part of the natural diet of fish, have been incorporated into feed formulations for various aquatic species, yielding promising outcomes. The black soldier fly, the yellow mealworm, and the common housefly are among the most promising insect species whose meals have been used to replace fish meal, fish oils, and soybean meal. A summary of recent findings by insect type and fish species follows hereafter (Table 4).

Wachira et al. [142] analyzed for a period of 20 weeks the suitability of black soldier fly larvae meal (BM) at three different dosages, 33, 67, and 100%, in partial or total replacement of fish meal (FM) on the growth of Nile tilapia (*Oreochromis niloticus*). The low BM dosage (33%) improved feed intake and weight gain compared to the control dietary treatment. Also, Piccolo et al. [143] studied the effect of *Tenebrio molitor* larvae meal (TM) supplementation (25% and 50%) in replacement of FM in sea bream (*Sparus aurata*) growth performance for 163 days, showing no negative effect on growth. The TM 25% diet gave the best results in terms of final body weight, weight gain, and feed conversion rate (FCR). Two recent comprehensive and systematic analyses of data on the impact of insects in aquafeed suggested a maximum threshold of 25–30% [144] or 30% [145] inclusion of insects in aquafeeds for uncompromised performance. Hua [145] underlined that the effects of insect meal levels on fish growth were influenced by dietary nutritional balance. Conversely, Khosravi et al. [146] documented, in juvenile rockfish (*Sebastes schlegelii*), a declining trend in weight gain and growth rates when TM inclusion levels surpassed 16%. Similarly, in another trial involving mandarin fish (*Siniperca scherzeri*) juveniles, it was observed that the growth rates and nutrient utilization efficiency increased when full-fat TM was included in their diets up to 20%. However, a decline in performance was noted at 30% of inclusion in the diet, in comparison to those fish that were fed an FM-based diet [147].

The evaluation of fish flesh quality and safety is crucial for consumers, and therefore, it is necessary to assess these parameters in fish that are fed insect-derived products [148]. Furthermore, the sensory quality of fish fillets is also significantly influenced by their diets [149]. The utilization of TM in the diet did not affect the water-holding capacity or texture characteristics of fillets obtained from blackspot sea bream (*Pagellus bogaraveo*), gilthead sea bream (*Sparus aurata*), and rainbow trout (*Oncorhynchus mykiss*) [143,150,151]. Replacing 25 and 50% of FM with TM in the diet of gilthead sea bream [143] or blackspot sea bream [150] did not alter the whole-body composition. Incorporation of maggot meal in diets of Nile tilapia (*Oreochromis niloticus*) at levels ranging from 25 to 100% FM replacement significantly increased hardness and reduced thaw loss in comparison to the control [152]. Fillet composition was not affected by the inclusion of BM at 15 to 45% of FM replacement in diets for European seabass (*Dicentrarchus labrax*) [153]. There were no significant differences in the texture properties of fillets of barramundi (*Lates calcarifer*) fed diets supplemented with tuna hydrolysate and BM (50 to 100 g/kg insect meal inclusion levels) [154].

The fatty acid profile of fish flesh is of utmost importance for human health. Concentrations of unsaturated fatty acids are high in mealworm, house cricket, and housefly maggot meals (60–70%), and low in BM (19–37%) [155]. Secci et al. [156] reported the effects of BM supplementation at various inclusion levels (0, 25, and 50%) in rainbow trout. The findings indicated that the flesh quality of the control group exhibited high levels of PUFAs and monounsaturated fatty acids (MUFAs) in comparison to the 50% inclusion levels. However, the inclusion levels at 25% did not result in any change to the flesh quality. In a study conducted by Bruni et al. [157], it was observed that the inclusion of *Hermetia illucens* full-fat meal at 25 and 50% inclusion levels did not have any impact on the fillet quality of *Oncorhynchus mykiss*. Similarly, Caimi et al. [158] observed that defatted BM can be utilized at concentrations of up to 15% without altering the fillet fatty acid profile.

**Table 4:** Main effects of insect meal inclusion as a supplementation or ingredient in fish diets.

Insect ^1^	Dietary Level	Animal Species, Category	Main Results vs. Control Diets	Reference
BM	0, 16.8, 33.6, and 57.1%	Nile tilapia (*Oreochromis niloticus*)	Feed intake and weight gain increased (up to +15% growth), while FCR ^2^ and survival were unchanged.	[142]
BM	0, 6.5, 13, and 19.5%	European seabass (*Dicentrarchus labrax*)	Up to 19.5% BM inclusion did not affect growth, fillet proximate composition, or overall quality. BM reduced fillet lipid peroxidation and oxidative stress indicators, with minimal effects on fatty acid profile and shelf-life.	[153]
BM	0, 20, and 40%	Rainbow trout (*Oncorhynchus mykiss*)	Fillet pH, colour, shear stress, and water-holding capacity were unaffected. BM 40% increased saturated fatty acids and reduced PUFA/MUFA, while BM 20% showed intermediate values.	[156]
BM	0, 10.5, and 21%	Rainbow trout (*Oncorhynchus mykiss*)	Growth and fillet quality were unaffected by BM inclusion. Fillet FA profile and key n-3 PUFA (EPA, DHA) were maintained, confirming good nutritional quality.	[157]
BM	0, 3, 6, 9, 12, and 15%	Rainbow trout (*Oncorhynchus mykiss*)	Growth, digestibility, and fillet physical traits were unaffected up to 15% inclusion. Higher BM levels increased SFA and MUFA and reduced PUFA.	[158]
MM	0, 9, 18, 27, and 36%	Nile tilapia (*Oreochromis niloticus*)	Up to 18% MM maintained growth, feed utilization, and flesh quality; 36% MM reduced survival and growth.	[152]
TM	0, 25, and 50%	Gilthead sea bream (*Sparus aurata*)	Diet 25% improved growth, FCR, and protein efficiency, while diet 50% reduced protein and lipid digestibility and dressed yield.	[143]
TM	0, 8, 16, 24, and 32%	Juvenile rockfish (*Sebastes schlegelii*)	Growth and protein retention improved up to 16% TM inclusion, then declined at higher levels. No changes in body composition or amino acid profile. Up to 16% TM is recommended, as 32% maintained similar performance but with reduced growth efficiency.	[146]
TM	0, 10, 20, and 30%	Juvenile mandarin fish (*Siniperca chuatsi*)	Growth and feed efficiency improved up to 20% inclusion, then declined at 30%. Fillets showed higher SFA and MUFA and lower n-3 PUFA compared to the control.	[147]
TM	0, 21, and 40%	Blackspot sea bream (*Pagellus bogaraveo*)	Growth and feed efficiency were unaffected by TM inclusion. Fillet texture and composition remained unchanged. Increasing TM reduced n-3 (EPA) and raised n-6 (linoleic acid), worsening the n-3/n-6 ratio and lipid health indexes.	[150]
TM	0, 25, and 50%	Rainbow trout (*Oncorhynchus mykiss*)	Growth, morphology, and fillet quality were unaffected. Fillet proximate composition remained stable, but TM increased C16:0, C18:1n9, and C18:2n6, while reducing EPA, DHA, PUFA/SFA, and n-3/n-6 ratios.	[151]

^1^ Insect species: BM: black soldier fly larvae meal; MM: maggot meal; TM: *Tenebrio molitor* larvae meal; ^2^ FCR: feed conversion ratio.

#### 3.3.2. Insects in Poultry: Performance and Product Quality

According to their feed preferences, digestive anatomy, and nutritional strategies, chickens and turkeys should not be considered as granivores but rather as omnivorous species [159]. In poultry farms, chickens with access to outdoor areas voluntarily consume insects in all stages of life [160], indicating that they are part of their natural diet [161]. This innate attitude of poultry species stimulated interest toward the use of insect protein meals in commercial poultry nutrition in replacement of traditional protein feedstuffs, particularly soybean meal. A summary of recent findings by insect type and poultry category follows hereafter (Table 5).

##### Broiler

Black soldier fly larvae meal is the most widely tested product in poultry nutrition [162], with the first attempts to evaluate its role as a feed additive dating back to the 1970s. Dabbou et al. [163] and Schiavone et al. [164] investigated the effects of graded dosages of partially defatted BM (0, 5, 10, and 15%) in broiler chicken diets. At slaughtering (35 d), the highest body weight was observed in broilers receiving the diet with 10% BM, while 15% inclusion impaired feed conversion ratio [163] as well as breast muscle yield [164]. Most of the breast meat quality traits were only marginally affected by the dietary treatments; however, broilers fed on diets with 15% BM yielded meat with the highest amount of MUFA and the lowest PUFA content, resulting in an imbalanced ratio between the two fatty acid families [164]. Such variation in the meat fatty acid profile is related to the BM richness in saturated fatty acids (mainly lauric acid, C12:0). According to these results, the authors concluded that BM can be included in broiler diets up to 10% without adverse effects on productive performance and product quality. On the other hand, Onsongo et al. [165], feeding broiler chickens diets containing black soldier pre-pupae meal at dosages from 0 to 15%, did not observe significant variations in performance parameters, processing yields, and meat quality traits. Mazlan et al. [166] found that replacing soybean meal with 10% BM had no adverse effect on the growth performance of broilers and reduced heat stress and pathogenic intestinal bacteria count. Other studies reported that partially defatted BM at dosages of 5% could be well tolerated by broiler chickens [167,168], while greater inclusion levels (around 8–10%) reduced average daily gain but had no remarkable effects on feed conversion ratio [167]. Finally, the dietary administration of full-fat BM to partially (50–75%) or completely replace soybean meal (tested dosages: starter, 20–40%; grower, 17–34%; finisher: 13–27%) compromised the growth performance and processing yields of Ross 308 male chickens [169]. Bovera et al. [170] pointed out that TM can represent a valid alternative to soybean meal in diets for growing broilers. Indeed, the Authors reported no adverse effects on the growth performance and product quality traits when soybean meal was completely replaced with TM (dosage around 30%) in diets for male Shaver brown chickens from 30 to 62 days of age. Biasato et al. [171] stated that increasing dosages of TM (5, 10, and 15%) can enhance body weight and feed intake but can impair feed conversion ratio, especially at the highest tested dosage. The use of TM at 7.5% of the diet did not affect the productive traits of a medium-growing genotype raised in free-range conditions [172]. Other studies reported that TM can be included up to 8% achieving either similar or better performance than feeding conventional diets [173,174].

Recently, some meta-analysis models have been obtained to better elucidate the effects of insect meal inclusion in broiler diets. Dalmoro et al. [175] indicated that insect meal inclusion above 10% can impair the growth performance of broiler chickens and that broilers fed TM presented higher daily weight gain compared to those receiving BM. On the other hand, Martínez Marín et al. [176] pointed out that a dietary inclusion up to 15% is not expected to negatively impact on growth performance of broilers, provided that the metabolizable energy and amino acid supply of insect-based diets is similar to those using conventional protein sources.

##### Laying Hens

In laying hens, Maurer et al. [177] reported no significant effect on egg deposition, feed intake, and egg quality traits by feeding Lohmann Selected Leghorn hens diets containing 12 or 24% partly defatted BM. Similarly, the use of meals from full-fat black soldier larvae and pre-pupae at 10% of the diet determined similar performance and egg quality parameters compared to a soybean-based diet [178]. Finally, Marono et al. [179] evaluated the total replacement of soybean meal with BM in diets for Lohman Brown hens from 24 to 45 weeks of age (i.e., 17% BM inclusion). Overall, the administration of the insect-based diet determined a better feed conversion ratio but reduced egg deposition rate, feed consumption, egg weight, and total egg mass. The results reported by Sedgh-Gooya et al. [180] suggest that 2.5 or 5% TM inclusion could enhance egg production, egg mass, and feed conversion ratio in Bovans White laying hens. Another study indicated that 5% TM did not alter egg deposition rate in Lohmann Brown laying hens, even though it reduced egg weight while increasing eggshell thickness and breaking strength [181].

##### Turkey

The number of published manuscripts evaluating the dietary inclusion of insect meals in turkeys is rather limited. Kozłowski et al. [182] evaluated the effects of the on-top administration of 0.3% full-fat meal from either mealworms or black soldier fly in diets for young turkeys. The results highlighted that the use of both insect meals did not affect the growth performance at 28 days of age, even though some beneficial effects were observed on oxidative and inflammatory parameters. In another study [183], a full-fat BM was included in the diets for turkey poults (i.e., up to 28 days of age) at dosages of 5, 10, and 15% in partial substitution for soybean meal. The Authors observed that increasing inclusion levels of BM linearly improved feed conversion ratio and tended to increase body weight and daily weight gain. Despite the limited number of animals used in the trial, Lalev et al. [184] concluded that 10% inclusion of either defatted or full-fat BM in diets for female turkeys from 56 to 130 days yielded acceptable productive performance and product quality.

**Table 5:** Main effects of insect meal inclusion as supplementation or ingredient in poultry diets.

	Insect ^1^	Dietary Level	Animal Species, Category	Main Results vs. Control Diets	Reference
**Broiler**	Partially defatted BM	0, 5, 10, and 15%	Ross 308	Growth and FCR ^2^ improved up to 10% inclusion but declined to 15%.	[163]
	Partially defatted BM	0, 5, 10, and 15%	Ross 308	Live and carcass weights improved up to 10% inclusion. Higher BM levels increased meat protein and SFA/MUFA while reducing PUFA and moisture.	[164]
	BM	0, 5, 10, and 15%	Cobb 500	Feed intake, growth, FCR, and meat sensory traits were unaffected.	[165]
	BM	0, 5, and 10%	Cobb 500	Growth performance and mortality were unaffected. BM up to 10% reduced heat stress and pathogenic intestinal bacteria count.	[166]
	BM	0, 15, and 30% replacement of soybean meal protein	Ross 308	15% replacement maintained growth, feed intake, gut health, and carcass traits. At 30% replacement, body weight decreased.	[167]
	BM	0, 50, 75, and 100% replacement of soybean meal protein	Ross 308	Growth and carcass quality were maintained at 50% replacement but declined at 75–100%. Higher inclusion reduced body weight, worsened FCR, and lowered meat juiciness and taste intensity.	[169]
	TM	0 and 29.7%	Shaver brown	Growth and carcass traits were mostly unaffected; FCR improved with TM.	[170]
	TM	0, 5, 10, and15%	Ross 708	Low TM inclusion improved body weight and feed intake. High inclusion (TM15%) reduced feed efficiency, indicating moderate levels are preferable.	[171]
	TM	0 and 7.5%	Label Hub- bard hybrid	Growth, welfare, hematological, and serum parameters were unaffected.	[172]
	TM	0, 2, 4, and 8%	Ross 308	Body weight and average daily gain, and FCR increased with TM, with optimal growth at 4% inclusion.	[174]
**Laying hens**	BM	0, 12, and 24%	Lohmann Selected Leghorn	Egg production, feed intake, yolk, and shell weights were unaffected. No health, plumage, or mortality issues were observed.	[177]
	BM larvae and pre-pupae	0 and 10%	Julia	Feed intake and egg-laying rate were unaffected. Egg weight and shell thickness increased in pre-pupae-fed hens.	[178]
	BM	0 and 17%	Lohman Brown	FCR improved with BM, but feed intake, egg weight, and total egg mass were lower.	[179]
	TM	0, 2.5, and 5%	Bovans White	TM improved egg production, egg mass, and FCR without negative effects on health or egg quality	[180]
	TM	0 and 5%	Lohmann Brown	TM did not alter egg deposition rate, but reduced egg weight while increasing eggshell thickness and breaking strength.	[181]
**Turkey**	BM and TM	0.3%	Young turkey	Growth performance at 28 days of age was not affected by both BM and TM; some beneficial effects were observed on oxidative and inflammatory parameters.	[182]
	BM	0, 5, 10, and 15%	Young Hybrid Converter turkey	Inclusion of BM improved gut health, microbial activity, and FCR without affecting growth.	[183]
	BM	0 and 10%	Female turkey	Enhanced growth and feed efficiency without negative effects on carcass or immune health.	[184]

^1^ Insect species: BM: black soldier fly larvae meal; TM: *Tenebrio molitor* larvae meal; ^2^ FCR: feed conversion ratio.

### 3.4. Camelina Sativa By-Products

Camelina sativa is a protein and oilseed crop that belongs to the Brassicaceae family [185]. Its cultivation dates back to the Bronze Age and, although grown for agricultural purposes in Europe until the mid-twentieth century, there has been a recent renewed interest in this plant, especially for biodiesel, fuel, and oil production [186,187]. Camelina sativa has low productivity, but its seeds contain about 40% oil, with a high proportion of n-3 PUFA. Camelina cakes and meals may represent alternative and cheap feedstuffs to be included in the formulation of poultry diet [187,188,189,190]. Indeed, they are characterized by the presence of residual oil (from 5 to 23%), containing about 30% α-linolenic acid, together with a high (30–35%) crude protein and essential amino acids content (15–18%, on as fed basis) (i.e., leucine, valine, lysine, phenylalanine, isoleucine) [187,189]. However, its use in poultry feeding is currently limited by the presence of plant secondary metabolites, such as glucosinolates and sinapine, having antinutritional properties [187,191,192]. Several studies evaluating the effect of different inclusion levels of camelina by-products in animal feeding pointed out its potential application in the formulation of the diet for both pork and poultry, at least up to certain dosages, without negative effects on the growth performance as well as on the meat quality [188,190,193,194]. However, when the available literature is examined, contradictory results are reported. These may likely be attributed to the variability existing in the quality of the camelina seeds, which may be related to the different growing and climatic conditions, as well as to the different oil extraction methods [187,191].

#### 3.4.1. Broiler

The dietary inclusion of camelina cake (up to 5%) during the grower and finisher phase did not affect the growth performance of broiler chickens [195]. On the other hand, when included at higher level (e.g., >10%) during the grower and finisher phases or in the diet for chickens up to 21 days of age, camelina cake exerted a detrimental effect on the growth performance of the birds (i.e., body weight, feed intake) for the lower ability of chicks and poults to digest camelina cake likely due to the limited efficacy of endogenous enzymes in the early development phases [195,196]. As for the effects on meat quality, the camelina inclusion in the poultry diet resulted in remarkable changes concerning the fatty acid profile of both breast and thigh meat. A significantly lower saturated and monounsaturated fatty acids content, with the latter being predominantly determined by the reduction in oleic acid, and a higher n-3 PUFA content were observed in camelina-fed birds [187].

#### 3.4.2. Laying Hens

Few studies were performed to test the effects of camelina cake inclusion on hens’ productivity. Cherian et al. [197] observed a significant reduction in egg production when camelina was included at 15%, while dosages up to 10% did not alter this productive trait. However, the same Authors reported significant effects on the quality traits of egg components (yolk weight, colour, and yolk/albumen proportion) for 10 and 15% dosages. Therefore, optimal inclusion levels could range from 5 to 10%. Aziza et al. [198] observed that 10% camelina can increase egg production in brown layers, even though crude protein and metabolizable energy digestibility were reduced. Finally, Lolli et al. [199] found that camelina inclusion up to 20% of the diet did not affect feed intake, egg productive performance, and welfare, while providing some benefits on eggshell breaking strength as hens become older.

## 4. Alternative Forages to Increase Protein Self-Sufficiency

### 4.1. Whole-Plant Soybean Silage

#### Agronomic and Nutritional Features

The diversification of crop rotations, including perennial or rotational legume forages are management strategy that can improve the sustainability of dairy farms [200]. The inclusion of legume forages into agricultural rotations allows for reducing N fertilization, thus GHG emissions, improving soil properties in terms of organic C, N, and P availability, and reducing the risk of production losses caused by weeds, pests, and diseases [201]. Being a short-season summer annual crop, differently from other high-protein forages such as lucerne, soybean can be inserted in a flexible rotation with other non-leguminous crops, especially maize, capable of maximize the benefits of N fixation [202] and in succession to different winter cereal crops (wheat, barley, triticale, etc.) to balance the self-production of the farm in terms of dietary energy and protein. In a dairy cow livestock system, self-production of whole-plant soybean silage (including stems, leaves, pods, and seeds) can increase protein self-sufficiency and can represent a valid strategy to reduce GHG and enhance the sustainability and profitability [203,204]. Forage soybean cultivars are generally taller, have higher leaf and stem DM, higher DM yield, and are later maturing compared to oilseed cultivars [205,206]. High variability in DM productivity has been reported, with average yields ranging from 2.4 [207] to 13.9 t/ha [202] in the USA and from 5.4 [200] to 9.2 t/ha [208] in Italy.

The different yields can be attributed to the different area, cultivar, seed density (row spacing), and stage of maturity at harvest. The DM yield of the whole soybean plant can be maximized by harvesting the soy between the reproductive stages R6 and R7 [207]. After stage R7, the leaves degenerate quickly, leading to a decrease in DM yield. The quality of the forage does not significantly decrease with advancing maturity due to the changes in the proportions of the stem, leaf, and pod fractions, the transfer of nutrients to the grain, and the increasing concentration of lipids in the seeds [209]. In this regard, neutral detergent fibre (NDF), acid detergent fibre (ADF), and acid detergent lignin (ADL) concentrations of the whole plant increase from R1 to R5 and decrease after R5, while CP concentration decreases from R1 to R3, is constant from R3 to R5, and increases after R5 [207]. These trends are confirmed by the values obtained by INRA [210] (Table 6).

The feasibility of ensiling soybean forage and obtaining qualitatively valid products has been questioned for several reasons [211]. This is mostly due to the high buffering capacity (caused by the high content of CP, oil, and ash), low water-soluble carbohydrate (WSCs) content, and insufficient epiphytic lactic acid bacteria (LAB) count of fresh soybean. However, despite the characteristics mentioned above, some studies demonstrated that ensiling the whole-plant soybean leads to a product with good nutritional characteristics that fits well with the diet of dairy cattle [206,208,212].

The choice of the proper soybean variety is the first step to obtaining a good silage. For example, Tabacco et al. [213] observed that low-size plants, compared to medium-tall size ones, had higher CP (24.4 vs. 20.0% on DM), lower ADL (6.4 vs. 9.0% on DM) contents, and higher in vitro NDF digestibility (IVNDFD) (51.5 vs. 46.2%) when harvested at R7 and after 200 days of ensiling. Moreover, several strategies have proven to be useful in improving the soybean silage quality. Some examples of these strategies are the field wilting of the forage prior to ensiling in order to increase the DM content [203], the addition of LAB alone or in combination with molasses [214] or chitosan [215] to promote the pH decrease and inhibit the proliferation of undesirable microorganisms, the mix of soybean with other forages rich in WSC such as corn or sorghum [216], and the addition of gallic acid to protect protein from degradation and promote the ensiling process [211].

In Table 7, it is possible to compare the chemical composition of three of the most common whole-plant legume silages (lucerne, pea, and soybean silages) [217].

Soybean silage has a CP content similar to pea silage but lower compared to lucerne silage, and the rumen-undegradable protein (RUP) percentage of soybean silage is the lowest among the silages presented. Moreover, soybean silage has less NDF and WSC contents, but higher starch and EE percentages compared to both lucerne and pea silages. Although soybeans generally have a lower annual yield compared to lucerne, it has to be remembered that soybean is a short-season summer annual crop that can be inserted in rotation with other non-leguminous crops, thus allowing for maximizing the production of plant material per hectare, with good yields in terms of both CP and energy. The IVNDFD reported in Table 7 is in line with the one found by Spanghero et al. [203] for soybean harvested at stage R6. In their work, Spanghero et al. [203] also studied IVNDFD of soybean silages harvested at different stages of maturity (R4, R5, and R6) and of their different plant components (leaves, pods, and stalks). Moreover, Spanghero et al. [203] observed, for the whole-plant soybean silage, an increase in IVNDFD from 32 to 39 and to 47% from R4 to R5 and R6, respectively. The explanation given by the Authors was that with advancing maturity, the proportion of pods (the plant components with the highest IVNDFD) on the whole plant increased, thus resulting in a greater IVNDFD of the whole-plant silage. In addition, also the in vitro CP degradability increased from 39.1 to 54.8% from R4 to R6 [203]. These results, together with the higher yield and the different chemical composition, suggest that it is preferable to harvest whole-plant soybean for silage at rather advanced stages (R6–R7) to obtain a greater DM yield, with higher percentages of protein and lipids, and more degradable at rumen level.

To the best of our knowledge, only four in vivo studies tested whole-plant soybean silage in the diet of lactating dairy cows. In the study of Rota Graziosi et al. [208], a control diet was compared to a diet including soybean silage (12.4% of total diet DM) in partial substitution of soybean meal (−35%). In Silva et al. [218], soybean silage was included at 8% of total diet DM, replacing 16% of maize silage present in the control diet. Ghizzi et al. [212] tested the inclusion of soybean silage at 0, 8, 16, and 24% of total diet DM, partially substituting maize silage. Lastly, Vargas-Bello-Pérez et al. [206] compared two diets with 36% of total DM coming either from soybean silage or lucerne silage. In three of the four studies mentioned above, the inclusion of soybean silage induced a reduction in DM intake (DMI), possibly due the higher NDF content and the higher proportion of long particles in the soybean silage diets [206,212,218]. Moreover, Ghizzi et al. [212] and Rota Graziosi et al. [208] observed that the inclusion of soybean silage reduced DM, OM, and NDF digestibility. The strict correlation between NDF digestibility and DMI is a consolidated phenomenon; in 1999 Oba and Allen [219] observed that an increase in NDF digestibility of one percentage point was linked to an increase in DMI of 0.17 kg.

The results regarding milk yield and dairy efficiency (kg milk/kg DMI) are not consistent. Rota Graziosi et al. [208] and Silva et al. [218] did not observe any difference regarding milk and FPCM yields, and dairy efficiency between the soybean silage diet and the control diet. Vargas-Bello-Pérez et al. [206] observed a reduction only in milk yield with the soybean silage diet compared to the lucerne silage diet. Ghizzi et al. [212] observed a linear reduction in milk yield, fat and protein corrected milk (FPCM) yield, and dairy efficiency as the inclusion level of soybean silage increases.

The inclusion of soybean silage could also influence milk N compounds. Milk urea nitrogen (MUN) was increased in the trial of Rota Graziosi et al. [208] and Vargas-Bello-Pérez et al. [206]. The lower milk protein percentage and the higher MUN found by Rota Graziosi et al. [208] with the soybean silage diet may have been caused by an unbalanced ratio of protein/energy provided with the diet. In this regard, due to the high protein degradability of legume silages in the rumen, increasing the amount of rapidly fermentable carbohydrates is recommended in order to enable the dietary N to be incorporated into microbial protein more efficiently [220]. The unbalanced ratio of protein/energy provided with the soybean silage diet could be the reason for greater N fecal and urinary excretions, which led to a lower N use efficiency found by Rota Graziosi et al. [208], contrary to the results of Silva et al. [218] which found no differences in terms of N balance between the soybean silage and the control diets. In synthesis, the results of these four in vivo studies suggest that whole-plant soybean silage can be included in the diets of lactating dairy cows without relevant negative effects on the performance of the animals if the diets are well balanced, especially considering the ratio and the rumen degradability of the protein and energy dietary components. In another study, Rota Graziosi et al. [204] performed a Life Cycle Assessment (LCA) study on the environmental impact of milk production systems characterized by different diets of lactating cows, including different sources of soybean. The use of soybean silage can reduce the global warming potential (GWP) of the diet and the environmental impact of milk production, due to the reduction in soybean meal inclusion. When compared to lucerne hay, the production of soybean silage resulted in higher GWP, marine eutrophication and human toxicity (the potential to cause harm to human health through exposure to toxic chemicals emitted during the life cycle of a product or process); however, soybean silage resulted a valuable option to reduce the environmental impact of milk production if the functional unit is referred to the land, by maximizing DM and CP yields per hectare if grown in succession to different winter crops [204].

### 4.2. Tef (Eragrostis tef)

Tef (*Eragrostis tef*, Poaceae) is a neglected warm-season C4 plant classified as intermediate between tropical and temperate grasses. It is traditionally grown in Ethiopia and Eritrea as a cereal crop, but it has also been grown as a grain and forage crop in the Americas, Asia, Australia, and the Middle East [221]. Due to tef’s great genetic diversity, it is believed to be a promising plant for developing accessions that could be suitably adapted to any geographical region [222].

#### 4.2.1. Proximate Composition and In Vitro Trials

Proximate composition of tef as forage has been occasionally evaluated mainly to address the CP, NDF, and ADF content of some varieties. The CP content was reported to vary between 89 g/kg DM [223] to 262 g/kg DM [224].

This great variability may be due to the different genotypes under investigation, but also due to different phenological stages at mowing, cutting number, or agronomic management. As an example, in some agronomic trials carried out in Oregon (USA), Roseberg et al. [225] reported the CP content of six genotypes of tef mowed at the early-heading stage (first cut) to be variable from 138 g/kg DM (cv. Pharaoh) up to 150 g/kg DM (cv. VAT-1). However, depending on the seeding date, these authors obtained tef plants with different CP content. In particular, two genotypes (namely, Pharaoh and VAT-1), seeded on mid and early June, respectively, had similar CP content (146.0 g/kg DM), but quite different agronomic yield (158 kg/ha and 487 kg/ha, respectively, for VAT-1 and Pharaoh). At the latest seeding time (June 23rd), the two genotypes performed quite differently with a lower yield and a higher CP content for the VAT-1 genotype (675 kg/ha and 164 g/kg DM, respectively) than for Pharaoh (897 kg/ha and 132 g/kg DM, respectively) [225]. Roseberg et al. [225] observed a poor correlation (r = 0.14) between agronomic yields and forage CP contents, apart from the genotypes Dessie (r = 0.99) and Tiffany (r = 0.69). Strong correlations (r = 0.84, and r = 0.80) have been observed between agronomic yield and NDF (420–602 g/kg DM), or ADF (330–367 g/kg DM) data.

The effect of nitrogen application (from 0 to 112 kg N/ha) in connection with cutting order (first vs. second) was studied on fodder production of tef (cv. Corvallis) by Hunter et al. [226]. As expected, there was a strong relationship (r = 0.998) between N application rates and the average CP contents of tef at first cutting (from 121.5 g/kg DM to 185.9 g/kg DM), but also for the second cutting (r = 0.995; CP from 97.9 g/kg DM to 191.5 g/kg DM). Interestingly, the data of Hunter et al. [226] also showed a possible interaction of nitrogen application rate and cutting order, favouring the constancy of the CP content passing from the first to the second cut, but only for the highest N application rate.

In a two-year study in Minnesota (USA), DeBoer et al. [224] evaluated the composition of two tef genotypes (namely, 6010 and Summer Lovegrass) according to the cutting order (first and second cuts) and the phenological stage (vegetative vs. mature) at mowing. The CP content (210 g/kg DM and 178 g/kg DM on average for the 6010 and Summer Lovegrass genotypes, respectively) was lower in forages mown at maturity (−14 and −16%, respectively, on average) with respect to the preceding phenological stage (vegetative), but it was particularly relevant in the occasion of the second cuttings (−22% for both genotypes). The NDF (529 g/kg DM and 611 g/kg DM, for 6010 and Summer Lovegrass, respectively) and the non-structural carbohydrates (71 g/kg DM and 101 g/kg DM, respectively) contents did not show any clear association with the cutting order, also in connection with the phenological stage at mowing.

A moderate role of the genotype on the IVNDF degradability (496.6 vs. 518.1 g/kg for cv Moxie and cv. Dessie, respectively) has been observed by Saylor et al. [227].

#### 4.2.2. Feeding Trials

Some feeding trials have been carried out in the USA to assess the suitability of tef hay for dairy cows [228,229] and beef cattle [230,231], with promising perspectives to substitute high-water-demanding forages such as alfalfa or corn silage. As an example, during a 54-day trial, Saylor et al. [228] assessed the productivity of multiparous Holstein cows fed on a diet including tef hay as the sole forage in comparison to counterparts fed on a diet with corn silage/alfalfa mix, as the main forage component. The tef-fed cows did not show different daily DMI with respect to the control ones, and no difference in milk production, milk fat, and lactose concentrations was observed between the two groups as well. At the same time, no changes in body condition score, body weight, total-tract DM or NDF digestibility were observed in the tef-fed cows compared to the controls.

In a 56-day trial with backgrounding cattle, Ream et al. [231] studied the effects of feeding tef (cv. Moxie) hay (65% of the diet DM), harvested at phenological stages corresponding to early-heading (EH, CP 144 g/kg DM; NDF 478 g/kg DM) or late-heading (LH, CP 131 g/kg DM; NDF 513 g/kg DM), on nutrient intake and digestibility, ruminal fermentation characteristics, and growth performance in 114 continental crossbred beef steers (initial BW 258 ± 19 kg). Dry matter, OM, and nitrogen intakes were significantly higher in the EH tef-fed steers for which were also observed to have higher total-tract DM and nitrogen digestibility, in comparison to the LH tef hay-fed ones. Total ruminal short-chain fatty acids (SCFAs) were higher in ruminal fluid of EH tef-fed steers than the controls, but only after 21 and 42 experimental days. In comparison to the tef forage harvested lately, the EH tef-diet gave better results than LH tef hay in terms of total BW change (80.9 vs. 73.6 kg), and average daily gain (1.44 vs. 1.31 kg/head/day), suggesting that the stage of maturity of tef at mowing should be carefully considered in the optimization of grass-based feeding systems.

## 5. Conclusions

Reducing the environmental footprint of animal production can be achieved, among other strategies, by partially replacing soybean meal with alternative protein sources. This review summarizes the main nutritional and functional properties of some promising alternative protein sources, both as concentrate ingredients and as fodder, and their effects on animal performance and product quality.

Among plant-derived feeds, legume seeds and oilseed by-products such as camelina meal offer valuable nutritional profiles and agronomic advantages as low-input crops.

Microalgae and insect meals also represent sustainable and versatile feeds with potential benefits for animal health and welfare, although their large-scale use is still limited by cost and optimization of inclusion rates.

At the forage level, the integration of alternative crops (such as soybean silage, tef, and triticale–lupin mixture) can enhance on-farm protein self-sufficiency and improve the environmental performance of livestock systems.

Further research is required to validate these alternatives under different production contexts and to support the progressive substitution of soybean meal in animal feeding strategies.

## Figures and Tables

**Table 1:** Nutritional characterization (g/kg DM or otherwise stated) of the most commonly used legume seeds in animal nutrition vs. soybean meal.

Legumes	DM ^1^, %	CP ^2^	NDF ^3^	ADF ^4^	ADL ^5^	Starch	EE ^6^	Ash	GE ^7^, MJ/kg DM	References
*Vicia faba* var. minor	85–92	250–290	126–240	100–160	4–12	300–450	9–13	34–41	18–19.5	[19,20,21,22]
*Pisum sativum*	87.8	233	104–246	65	0.5	329–530	13	31	19	[20,22]
*Cicer arietinum*	92–95.5	255–284	123–154	46–47	0.5–1	332–356	45–46	34–39	18.8–19	[20,23]
*Lupinus albus*	91–92	309–454	189–217	96–184	27–49	13–74	70–106	44–68	8.0	[20,24,25,26]
*Lupinus luteus*	90.5–91.1	322–343	217–252	195–213	15–24	38–49	52–59	40–66	20.5	[27]
*Lupinus angustifolius*	90–90.5	277–303	220–312	236–276	29–37	45–101	33–46	47	20	[22,26,28]
Soybean meal	86.8–88.8	478–512 (44% CP) 536–568 (49% CP)	125–171 (44% CP) 88–122 (49% CP)	67–111 (44% CP) 45–69 (49% CP)	3–13 (44% CP) 1–7 (49% CP)	9 (44% CP) 11 (49% CP)	13–25 (44% CP) 11–23 (49% CP)	67–81 (44% CP) 68–78 (49% CP)	18.9–20.1 (44% CP) 194–200 (49% CP)	[29]

DM ^1^: Dry matter; CP ^2^: Crude protein; NDF ^3^: Neutral detergent fibre; ADF ^4^: Acid detergent fibre; ADL ^5^: Acid detergent lignin; EE ^6^: Ether extract; GE ^7^: Gross energy.

**Table 2:** Bioactive compounds of the most commonly used legume seeds in ruminant nutrition with potential antioxidant roles ^a^.

Legumes	*Vicia faba* var. Minor	*Pisum sativum*	*Cicer arietinum*	*L. albus*	*L. luteus*	*L. angustifolius*
Phenolic compounds	4.8–13 * (pod extract)	0.5–1.3 *	47 ± 18.4 **	227 ± 137.9 **	960 ± 203.9 **	679 ± 20.2 **
	2.9 * (whole seed)	152 ± 64.2 **	*§ 0.93–10.8			
	22.5 * (seed coat)					
	214 ± 120.6 ** (whole seed)					
- *Phenolic acids*	23–138 **	57–248 **	27–107 **	7.5 **	10–28 **	nd ***
- *Flavonoids*	6–245 **	6–57 **	0–21 **	130–465 **	706–1144 **	665–692 **
			148.5–302.1 ††† (whole seeds)			
- *Tannins*	2.1–7.4 †	4.69 †	3.78 †	3.1–7.7 !	2.2–2.7 !	1.3–1.6 !
Carotenoids	3.4–3.5 †††	5.41–28.19 ††	8.9–31.3 ††	8.9–12.7 **	6.1–6.3 **	17.2–62.5 **
Tocopherols	5.4–6.2 ††	6.54–13.9 ††	8.7–11.3 ††	922–1834 ††	1415–1773 ††	674 ††

* mg GAE/g; § according to different organic solvents (acetone, ethanol, methanol); ** mg/kg DM; *** not detected; †: g/kg DM; †† µg/g FW; ††† µg/g DM; ! mg/100 g. ^a^: supporting references: [34,35,36,37,38,39,40,41,42,43].

**Table 3:** Main effects of incorporation of *Spirulina* (SP) and *Chlorella* (CHL) as supplementation or ingredient in the diets of monogastric species.

	Microalga	Dietary Level	Category, Initial Body Weight and Age	Main Results vs. Control Diets	Reference
**Piglets**	*SP*	2–20 g/kg for 28 d	Weaning piglets, 3.7 kg, 11–12 d	No change in growth performance	[105]
	*SP/CHL*	1% as fed for 14 d	Weaned piglets, 9.1 kg, 28 d	Improved intestinal mucosa and nutrient digestibility. Positive effects of CHL on digestive disorders after weaning	[106]
	*SP/CHL*	385 mg/kg BW for 14 d	Weaning piglets 4.9 kg, 14 d; weaned piglets 9.04 kg	Increased weaning weight and reduced diarrhea occurrence	[107]
	*CHL*	5% as fed for 15 d	Weaning piglets 11.2 kg, 28 d	No change in growth performance and meat quality. Increased carotenoids and n-3 PUFA content, enhanced antioxidant activity, and hepatic lipid metabolism	[108]
**Fattening pigs**	*CHL*	5% as fed from 59.1 to 101 kg BW	Barrows 59.1 kg	No changes in growth performance, carcass, and meat quality traits. Increased lipid-soluble antioxidant pigments and n-3 PUFA of meat. Strong immunosuppressive effect, and increased hepatic content of n-3 PUFA	[109,110]
	*SP*	50 to 100% replacement of soybean, from 22 to 75 kg BW	Barrows 22 kg	No change in sensory attributes and physico-chemical parameters of meat.	[111]
**Sows**	*SP*	20 g/d for 29 weeks	Gilts 119.3 kg, 5.6 months	Moderate effects on growth performance, carcass, and meat quality of the offspring	[112]
**Broilers**	*SP*	40–80 g/kg as fed for 16 d	Male chicks 678 g, 21 d	No change in growth performance. Increased redness and yellowness of meat	[113]
	*SP*	100% replacement of corn (study 1), 6–21% DM for 18 days (study 2)	Chicks 3 d	Increased amino acids digestibility with 100% replacement; no changes until 16% replacement, decrease in performance and amino acid digestibility with 21% replacement	[114]
	*SP*	50% replacement of soybean for 35 d	Male chicks 1 d	SP increased pH and WHC and decreased metallic off-flavour of meat.	[115]
	*SP*	50 to 75% substitution of soybean for 35 d	Chicks 1 d	SP increased meat colour and lipid oxidation; no effects on sensory attributes.	[116]
	*CHL*	25 to 75 g/kg for 35 d	Chicks 1 d	CHL increased growth rate, immune response intestinal microflora status	[117]
	*CHL*	0.2 to 1% for 35 d	Chicks 45 g, 1 d	With 1% inclusion, better growth rate, feed conversion ratio, and immune status.	[118]
**Laying hens**	*SP*	1.5 to 2.5% for 4 weeks	Hens 1500 g, 63 weeks	No changes in production performance, increase in egg yolk colour with SP at 2.5%	[119]
**Rabbits**	*CHL*	200 to 500 mg/kg BW	Females, 935 g, 6 weeks	Increases feed intake, BW, carcass weights, and reduction in oxidative stress	[120]
	*CHL*	0.5 to 1.5 g/kg diet for 8 weeks	Males, 635 g, 5 weeks	No change in growth performance, reduced alanine transferase activity, increase in hematochemical and immune parameters	[121]
	*SP/CHL*	300 to 500 mg/kg diet for 8 weeks	Males, 665 g, 5 weeks	Improved growth, intestinal enzyme efficiency, blood health, and antioxidant parameters.	[122]

**Table 6:** Chemical composition (g/kg DM or otherwise stated) of early maturing and late maturing varieties of whole-plant soybean fresh forage at different maturity stages (adapted from INRA, [210]).

Maturity	DM ^1^ (%)	OM ^2^	CP ^3^	NDF ^4^	ADF ^5^	EE ^6^	GE ^7^ (kcal/kg DM)
Early varieties:							
Pod setting R3	17.2	895	174	461	326	25	4380
Seed setting R5	23.2	910	180	422	293	50	4460
Mature seeds R7	32.1	918	228	441	309	75	4700
Late varieties:							
Beginning of flowering R1	16.2	888	154	467	331	20	4350
Flowering R2	18.9	892	150	488	348	10	4370
Pod setting R3	20.8	897	169	469	332	25	4390
Seed setting R5	23.9	908	171	458	323	35	4450

^1^ DM: dry matter; ^2^ OM: organic matter; ^3^ CP: crude protein; ^4^ NDF: neutral detergent fibre; ^5^ ADF: acid detergent fibre; ^6^ EE: ether extract; ^7^ GE: gross energy.

**Table 7:** Chemical composition of lucerne, pea, and soybean silages (adapted from NASEM, [217]). The values are on a DM basis (g/kg DM), except where otherwise stated.

	DM ^1^ (%)	Ash	CP ^2^	RUP ^3^ (%CP)	NDF ^4^	IVNDFD ^5^ (%NDF)	ADF ^6^	ADL ^7^	Starch	WSC ^8^	EE ^9^
Lucerne silage	42.9	106	205	27.0	432	49.4	337	74	20	63	29
Pea silage	31.7	114	170	27.0	525	57.7	371	64	34	45	38
Soybean silage	37.5	101	180	21.0	453	47.7	356	78	45	43	43

^1^ DM: dry matter; ^2^ CP: crude protein; ^3^ RUP: rumen-undegradable protein; ^4^ NDF: neutral detergent fibre; ^5^ IVNDFD: in vitro 48 h NDF digestibility; ^6^ ADF: acid detergent fibre; ^7^ ADL: acid detergent lignin; ^8^ WSC: water-soluble carbohydrates; ^9^ EE: ether extract.

## Data Availability

No new data were created or analyzed in this study.
